# Healthcare professionals’ perceptions of childhood obesity in Iquitos, Peru: a qualitative study

**DOI:** 10.1186/s12913-022-07519-z

**Published:** 2022-02-10

**Authors:** Jo F. Lai, Joanne Clarke, Gilles de Wildt, Graciela Meza, Miriam A. Addo, Esme Gardiner, Divya Khanna

**Affiliations:** 1 College of Medical and Dental Sciences, University of Birmingham, Birmingham, UK; 2 Facultad de Medicina Humana, Universidad Nacional de la Amazonía Peruana, Iquitos, Peru

**Keywords:** Childhood obesity, Peru, Perceptions, Healthcare professionals, Qualitative

## Abstract

**Background:**

Childhood obesity is an urgent worldwide concern associated with increased morbidity in adulthood. Healthcare professionals (HCPs) are well placed to influence childhood obesity trends and implement interventions. English-language studies regarding HCPs’ perceptions of childhood obesity are limited to high-income countries. Peru is an upper-middle-income country with regional disparities in childhood obesity prevalence. This qualitative study aims to explore HCPs’ perceptions of childhood obesity in Iquitos, Peru, where prevalence is relatively low.

**Methods:**

Twenty-one HCPs with child healthcare experience were purposively recruited from two primary healthcare centres. Semi-structured, individual interviews were conducted with a translator and audio recorded. Transcribed data were analysed using thematic analysis.

**Results:**

Eight themes were identified and divided into four categories: (1) HCPs’ perceptions and attitudes towards childhood obesity (level of concern regarding childhood obesity, perceived consequences of childhood obesity); (2) Factors which HCPs perceive to be important in the development of childhood obesity (parental factors, contextual factors); (3) HCPs’ perceptions of their role in childhood obesity prevention and management (educating parents about childhood obesity, regular monitoring of child growth); and (4) Barriers and facilitators in childhood obesity prevention and management (in healthcare, in schools).

**Conclusions:**

HCPs had a low level of concern regarding childhood obesity in Iquitos and prioritised undernutrition. Parental factors were perceived to be the most influential in the development of childhood obesity. HCPs perceived themselves to have minimal influence due to prevailing positive views of excess weight and difficulties engaging parents. Educating parents about childhood obesity was felt to be essential to prevention and management although regular monitoring of child growth and home healthcare visits were viewed as useful additional measures. This study can help to inform the development of targeted public health strategies which are sensitive to local contexts and could prevent the upward childhood obesity trends evident elsewhere in Peru.

**Supplementary Information:**

The online version contains supplementary material available at 10.1186/s12913-022-07519-z.

## Introduction

The rising rate of childhood obesity is an urgent worldwide concern [1]. Childhood obesity is associated with increased morbidity in adulthood [2] and is therefore a major contributor to the mounting burden of non-communicable diseases [1]. Globally, an estimated 124 million children were classified as obese in 2016 and this number continues to rise [3]. Between 1975 and 2016, the prevalence of childhood obesity in 5 to 19-year-olds rose from 0.7 to 5.6% in girls and 0.9 to 7.8% in boys [3]. However, there is geographical variation in the rate at which prevalence is increasing [3, 4]. The upward trend in childhood obesity has plateaued in high-income countries but remains increasing in low and middle-income countries [3]. Regional disparities may reflect variations in cultural and societal influencing factors. For example, protective factors such as a lifestyle with high levels of physical activity as seen in Belgium, or risk factors such as the increase in food availability as seen in the USA [5]. Therefore, regional factors which may influence childhood obesity trends merit exploration to inform our understanding of this global health issue.

In Latin America, one in five under 20-year-olds are overweight or obese [6]. Peru is an upper-middle-income country [7] with the third highest prevalence of childhood obesity in Latin America [8]. Although the prevalence rate of childhood obesity in Peru has stabilised in recent years, there is significant variation in its distribution across the nation’s 25 regions, or ‘departments’ [9]. The highest prevalence rates are mostly in the coastal departments, including Peru’s capital, Lima. Among the three departments with the lowest prevalence rates is Loreto [9], the largest and northernmost department located in the Amazon rainforest. Its urban capital, Iquitos, is a large city only accessible by river or air. Different childhood obesity prevalence rates across Peru reflect different stages of the ‘nutrition transition’. Lima and a few other, mainly coastal, regions have completed the transition from a population with prevalent undernutrition to a population with prevalent obesity [10]. Most departments, including Loreto, are reportedly in the intermediary stage as they are experiencing the ‘double burden of malnutrition’ where both undernutrition and overweight or obesity, particularly in children, are a concern [10]. Therefore, decentralised nutrition policies have been recommended in Peru as policies dictated at a national level may not account for differences between departments [10].

Peru is committed to the Pan American Health Organization’s Plan of Action for the Prevention of Obesity in Children and Adolescents [11] and has implemented improvements to marketing regulations and school nutrition [12, 13]. However, as evidence underpinning policies are derived from studies outside of Peru [11] and local culture may introduce discrepancies from international standards [14], investigations exploring local perceptions are warranted in order to develop more targeted strategies.

Despite Peru’s trajectory towards nationwide completion of the nutrition transition, and thus a greater prevalence of childhood obesity [10], research is limited. A 2016 qualitative study investigated adolescent eating behaviours in Lima [15]. No additional English-language qualitative studies regarding childhood obesity in Peru could be identified from extensive literature searches. Therefore, further exploration into perceptions of childhood obesity is necessary to address this lack of qualitative literature and to contextualise future research or policies.

As providers of patient education and health promotion, healthcare professionals (HCPs) are well placed to influence childhood obesity trends and implement interventions. Their role is manifold and includes preventing, recognising and managing overweight or obesity in children. Input from HCPs is particularly important as childhood obesity is largely preventable and 50.7% of parents underestimate the weight status of their obese or overweight child [16]. Internationally, multiple studies have explored the perceptions of HCPs regarding childhood obesity. A 2018 meta-synthesis identified 13 qualitative studies on HCPs’ experiences of discussing child weight with parents [17]. The study found that barriers and facilitators were most frequently attributed to intra/interpersonal factors [17] demonstrating the value of investigations at the individual level. The importance of considering HCPs’ beliefs is reiterated by a 2009 systematic review of cross-sectional studies [18]. The authors identified a need for physician-targeted training based on their finding that doctors lacked confidence in their capacity to effectively manage childhood obesity [18]. Furthermore, a 2019 scoping review found that the views of HCPs regarding causal factors and effectiveness of interventions were an important barrier in adult obesity prevention services [19]. However, studies concerning the perceptions of HCPs in childhood obesity research are limited to high-income countries [17, 18]. Therefore, the transferability of their findings to Peruvian HCPs is limited and necessitates context-specific studies. It is valuable to conduct research particularly in areas which are transitioning from prevalent undernutrition, such as Loreto, as the changing needs of a region should be acknowledged and addressed in a timely manner to avoid the onset of prevalent obesity [20].

The aim of this qualitative study is to explore perceptions of childhood obesity in HCPs with child healthcare experience in Iquitos, Peru. This encompasses in-depth investigations into their personal views in addition to their experiences and perceptions of preventing or managing childhood obesity in their role. The aim will be achieved through the following objectives:

- To explore HCPs’ perceptions and attitudes towards childhood obesity
- To explore which factors HCPs perceive to be important in the development of childhood obesity
- To explore HCPs’ perceptions of their role in childhood obesity prevention and management
- To explore HCPs’ perceptions of barriers and facilitators in childhood obesity prevention and management

## Methods

### Study design

This study employs qualitative methods guided by grounded theory [21] to conduct and analyse semi-structured interviews with HCPs. A qualitative approach was most appropriate in order to explore participants’ experiences in-depth [21]. The study is reported in accordance with the Consolidated criteria for reporting qualitative research (COREQ) [22].

### Setting

Participants were recruited from two primary healthcare centres in Iquitos, a city located within the Amazon rainforest with approximately 440,000 residents, almost half of the total population of Loreto [23]. Within Peru’s four-tiered classification system for primary healthcare centres, both recruitment sites are category 4 establishments. This indicates provision of the highest level of specialist care available outside of hospitals [24] thereby providing access to a wide range of HCPs for recruitment.

Childhood obesity in the study area is relatively low compared with coastal Peru, which saw a transition from low to high levels. However, it is important to explore perceptions and factors in this setting that can be influenced by health promotion and other policy measures to mitigate the risk of a similar transition.

### Recruitment and sampling

Recruitment occurred during January and February 2020. Participants were sought via purposive sampling to include individuals in regular contact with the local paediatric population whilst maintaining a range of perspectives by involving various professions. Eligibility criteria were devised to access HCPs relevant to the study’s aims and for pragmatic reasons. Inclusion criteria were: (1) HCP with experience of working in child healthcare, (2) fluent in Spanish or English and (3) aged 18 or over. At the two recruitment sites, HCPs were approached in-person throughout the recruitment period. After a brief explanation about the study and the researchers’ background, interested HCPs were provided with a participant invitation letter and information sheet in Spanish. Those eligible and willing to partake in the study arranged a suitable interview time with the lead researcher. Only one individual, a psychologist, declined to take part. Recruitment ended upon reaching data saturation determined through data analysis which was performed alongside data collection.

### Data collection

Semi-structured interviews were conducted to enable an open-ended exploration into participants’ perceptions which allowed novel concepts to arise until data saturation was achieved. Interviews were individual and face-to-face to facilitate rapport building.

A topic guide (Additional file 1) was developed based on the research objectives and, to a limited extent, included prompts on topics derived from extant literature [17, 18]. The topic guide included questions on HCPs’ understanding of childhood obesity, their views of their roles as well as prevention and management strategies. A translated topic guide was piloted with an HCP to refine questions and identify potential practical issues. The pilot interview was not included in the data analysis.

All interviews were conducted by the lead researcher, a female undergraduate student trained in conducting in-depth interviews. Real-time interpretation was provided by an experienced female research translator who has supported previous qualitative studies. Both individuals had no prior relationship with any participants. As all participants communicated in Spanish, the interpreter mediated in all interviews for the English-speaking lead researcher who understood a basic level of Spanish. The interpreter was briefed on her duty to maintain confidentiality and translate as accurately as possible which was agreed in a signed statement.

Interviews took place in a private room at the healthcare centres and were audio recorded on a password-protected, encrypted device. At the start of each interview, written and verbal consent were obtained, and the participant self-completed a demographics questionnaire. Audio data for each interview was anonymised and produced three transcripts comprised of (1) verbatim English, (2) verbatim Spanish and (3) redacted translations of the Spanish with hesitations and faltering speech removed. The accuracy of translations was confirmed by an independent translator using transcripts from the first two interviews. Field notes were taken promptly after each interview to record non-verbal aspects and document initial reflections which included the researcher’s experience, decisions and interpretations [25]. In conjunction with reviews of transcripts and discussions with the wider research team, the field notes enabled the point of data saturation to be identified after the twenty-first interview as no new points were raised.

Seeking member validation was not practicable due to time constraints. Participants could stop the interview or refuse a question at any time and could voice general thoughts without prompting at the end of the interview. Participants could withdraw from the study up to 3 days post-interview without providing reason. No participants withdrew or refused questions and no repeat interviews were required.

All methods were carried out in accordance with the guidelines and regulations of the ethical bodies governing this study.

### Data analysis

An inductive thematic analysis was undertaken to identify themes using the transcripts as the primary source of data with additional context provided by field notes to aid data interpretation. This approach was structured according to Braun and Clarke’s six-phase guide to thematic analysis to ensure results were produced through a comprehensive and recursive interpretation of the data [26].

A constant comparative approach was adopted which considered new information alongside the existing data set [27]. This iterative process enabled unanticipated areas of interest to be acknowledged through appropriate adaptation of the topic guide during data collection, such as the inclusion of questions regarding socioeconomic factors after participants raised this issue.

Familiarisation with the data was achieved through the production of English transcripts by the lead researcher and furthered in the process of checking translations in the Spanish and translated transcripts. Transcribed data were coded in NVivo 12 software according to Saldaña’s two-cycle process whereby coding is undertaken twice to produce, firstly, descriptive codes and, secondly, more interpretive codes [28].

Preliminary findings were triangulated with two researchers, both undergraduate students conducting research concurrently in Iquitos. Each individual coded one transcript and discussed their analyses with the lead researcher prior to the second cycle of coding in order to provide multiple perspectives.

Themes were derived from the transcribed data via a coding tree consisting of descriptive codes which were built upon with interpretive codes and input from field notes. To comprehensively address the aim of the study, the themes were categorised according to the four research objectives.

## Results

### Participants

Twenty-one participants were recruited excluding one pilot interview. The average interview length was 42 min, ranging from 24 to 58 min. Professions spanned five broad groups which were: dentist, obstetrician, nurse, doctor and nutritionist. Nurses identified as either a technical nurse or licensed nurse, the latter holding more clinical responsibilities. Participant characteristics are summarised in Table 1.

**Table 1:** Summary of participant characteristics

Characteristic	Number of participants (Total = 21)
Age range	
20–29	8
30–39	6
40–49	5
50–59	2
Gender	
Female	16
Male	5
Profession	
Dentist	2
Assistant Obstetrician	1
Technical Nurse	7
Licensed Nutritionist	1
Licensed Nurse	5
Family Doctor	1
Obstetrician	3
Surgeon	1
Time qualified (years)	
< 1	4
1–5	5
6–10	5
11–15	3
16–20	2
21–25	1
26–30	0
31–35	1

### Findings

Eight themes were identified and were categorised according to the research objective which they best addressed. Salient points of each theme are summarised in Table 2.

**Table 2:** Summary of themes categorised according to the research objectives

**Category 1: HCPs’ perceptions and attitudes towards childhood obesity**		
1a	Level of concern regarding childhood obesity	• Childhood obesity is not a major concern in Iquitos • Undernutrition is a greater priority than obesity
1b	Perceived consequences of childhood obesity	• Long-term medical implications • Psychological consequences, particularly in adolescents
**Category 2: Factors which HCPs perceive to be important in the development of childhood obesity**		
2a	Parental factors	• Parents have the most influence • Positive views of excess weight prevail
2b	Contextual factors	• Availability of technology, affordable healthy foods and outdoor space • Perceived association with socioeconomic status
**Category 3: HCPs’ perceptions of their role in childhood obesity prevention and management**		
3a	Educating parents about childhood obesity	• Addressing parental misconceptions • Supporting the family as a whole
3b	Regular monitoring of child growth	• Key to recognising overweight or obese children • Enable interventions to be initiated
**Category 4: Barriers and facilitators in childhood obesity prevention and management**		
4a	Barriers and facilitators in healthcare	• Barrier – Lack of parental cooperation • Facilitator – Utilising home visits
4b	Barriers and facilitators in schools	• Barrier – Lack of interest from teachers and parents • Facilitator – Platform for education and government policies

### HCPs’ perceptions and attitudes towards childhood obesity

#### Level of concern regarding childhood obesity

All participants discussed childhood obesity from a pathogenic perspective and associated it with negative consequences. Although many had encountered cases of overweight or obese children, the majority believed that childhood obesity was not common nor increasing in Iquitos and had a low level of concern.

*“I don't think [childhood obesity] is increasing here in Iquitos, there may be cases of obesity in children, but it's not extreme”* (P9 – Surgeon)

This regard for childhood obesity as a lower priority was contrasted by a high level of concern for undernutrition, which was considered a more important health issue in children.

*“What you see most is child undernutrition, it's more relevant. Yes, there are obese children, but the greatest percentage are undernourished”* (P13 – Licensed Nurse)

Some participants believed that childhood obesity was increasing but maintained the perception that it is not currently a priority in Iquitos, a view shared by all participants. Comments on prevalence rates were often contextualised using other countries or Peru’s coastal regions to highlight that trends in Loreto were not concerning.

*“In other countries, such as the United States, there is a greater index of obesity compared to Loreto. I could highlight the undernutrition in Loreto compared to other countries. There is very little obesity here”* (P14 – Licensed Nurse)

*“I think other places attach more importance to childhood obesity than here in Loreto. Here, […] they are definitely putting childhood obesity aside”* (P20 – Licensed Nutritionist)

#### Perceived consequences of childhood obesity

Universally, discussions surrounding the implications of childhood obesity were approached in terms of long-term adverse health outcomes. Increased risk of diabetes and cardiovascular diseases in adulthood predominated the lists of medical consequences which were cited.

*“If we don’t recognise the problem of obesity in children, in the future this will become an adult who will suffer from metabolic diseases such as diabetes mellitus, hypertension and dyslipidaemia”* (P9 – Surgeon)

Reports of consequences which manifested in childhood were less common and included physical problems such as “*problems of their joints, […] support of their hips, their knees and their ankles”* (P19 – Licensed Nurse). Bullying from peers was mentioned by several participants, with a few also recognising some psychological consequences of teasing. Mental health issues resulting from being overweight were usually raised in relation to adolescents.

*“When you reach puberty, you realize that you are fat. Then come the social factors, the bullying. [Others] aggravate them because they’re fat, and that's when they first realise that they’re obese and start to become aware”* (P6 – Family Doctor)

### Factors which HCPs perceive to be important in the development of childhood obesity

#### Parental factors

The perception that parents possess the most influence in the development of childhood obesity was a pervasive theme in all discussions regarding contributing factors and prevention strategies. Parental influence was largely viewed negatively, as participants focussed on the detrimental impact of parents on their children, typically through their role in imparting unhealthy eating behaviours.

*“[Childhood obesity] depends a lot on the parents because they are the ones who live with them 24 hours a day and I think we should address them first so that they can help the child too”* (P13 – Licensed Nurse)

*“[Responsibility is] mostly the parents’, because they must guide and educate their children properly so that later they do not suffer from disease”* (P18 – Technical Nurse)

Parents were often criticised for either acting as poor role models to their children or conceding to their children’s requests for junk food.

*“Here at the facility, we see that the mother brings in a bottle of sugary drink, instead of bringing something healthy”* (P4 – Technical Nurse)

*“[Parents] say that they feed their children [certain foods] because their children want to eat that, for example, if the child says they want to eat chocolate, then they’ll give them chocolate”* (P7 – Obstetrician)

The sentiment that parents lacked awareness about healthy eating behaviours was commonly proposed to explain these bad practices.

*“Another important factor [in childhood obesity] is the lack of awareness in parents because they just want to give [children] food but they don’t care if they are receiving the right nutrients for their body”* (P7 – Obstetrician)

Notably, participants reported that there was a prevailing belief amongst parents that excess weight in children is desirable and signals health, which may account for the lack of interest in adopting healthier behaviours. HCPs perceived the lack of understanding about the pathogenicity of obesity to be widespread and a challenge to navigate.

*“A lot of the time, parents think wrongly of their kids that the fatter they are, the healthier they are”* (P14 –Licensed Nurse).

*“It is a challenge [to understand obesity] for us as professionals, as well as for the families, because mothers think that an obese child is healthy, when in fact it’s not”* (P5 – Licensed Nurse)

Although participants themselves viewed childhood obesity as a problematic health issue, one admitted that their own perception had been different previously.

*“We thought [obesity] was inherited, right? If the parent is fat, then [the child is] fat too. But now, over time, studies show that weight gain in children is pathological”* (P2 – Assistant Obstetrician)

In contrast to the negative attitude towards parents demonstrated in most interviews, one participant felt that parents contributed positively to child health.

*“In our city, you observe […] mothers who care about their children's nutrition and enrol their children in different holiday courses such as football, volleyball, basketball, etc. to always keep them active”* (P10 – Licensed Nurse)

#### Contextual factors

Beyond parental influences, participants raised a variety of issues which appeared to be a product of the wider context within which their patient population lived. The rainforest location of the city proved, for some participants, to be an influential factor in childhood obesity as it was thought to determine three contextual factors: (1) the availability of technology, (2) affordable healthy foods and (3) outdoor space for physical activity. Generally, the HCPs felt that Iquitos had limited access to the former two commodities whilst a few mentioned that there was a greater abundance of the latter.

Greater use of technology, such as televisions and phones, was perceived to accelerate the development of obesity in children by increasing sedentary behaviour. However, this was less of a concern in Iquitos compared to elsewhere.

*“Childhood obesity here is not so common mainly because we don't have the most advanced technology yet [...] here, children are not totally focused on technology, you rarely see children with their tablets, phones or TVs, they prefer to go out and play in the streets”* (P10 – Licensed Nurse)

Another location-dependent factor recounted by some participants was the high prices of fruits and vegetables. This generated a financial barrier to change according to some HCPs who empathised that eating healthier foods may incur an economic cost to families.

*“Here in our region, carbohydrates are the most consumed, we don't consume fruits or vegetables. Sometimes because of the cost, it’s a little bit inaccessible”* (P2 – Assistant Obstetrician)

*“Parents don’t have the financial freedom to buy the right foods”* (P18 – Technical Nurse)

Most reports of physical activity levels further suggest that the environment which Iquitos provides children may have protective elements.

*“One of the advantages we have in Iquitos is that we don’t live in an environment with as much risk or danger as the big cities on the coast. Here, the children still go out to play, […] they have more free spaces than other cities”* (P9 – Surgeon)

The micro-context of the family unit was also presented as a potential factor which determined if a child became obese. Many participants associated a family’s socioeconomic status with the likelihood of developing childhood obesity. However, participants differed on whether it was low or high socioeconomic status which increased the likelihood of childhood obesity.

*“Economic aspects prevent you from eating healthy fruits or vegetables as they are expensive in this region”* (P6 – Family Doctor)

*“Financial accessibility to have video games […] makes children today remain more inactive”* (P9 – Surgeon)

Some believed that families of higher socioeconomic status had greater access to food and so they were more likely to have overweight children.

*“People who have a little more money […] buy junk food to please the child”* (P16 – Technical Nurse)

*“I think [childhood obesity is common] in the middle class because it also occurs in the class that has more wealth”* (P13 – Licensed Nurse)

This view was usually compounded by the perception that undernutrition was more commonly seen in families with lower socioeconomic status, which may contribute to the lay perception that having overweight children is desirable.

*“[Obesity occurs] from the middle class and up and the lower group is where we attend patients with child undernutrition”* (P1 – Dentist)

*“The common thing you see is that the father of the family doesn’t have a job. If they don’t have a job, how are they going to feed the child”* (P17 – Obstetrician)

Meanwhile, a relationship with socioeconomic status in the other direction was also posited. Some participants suggested that higher socioeconomic groups could be healthier because *“they can buy better food, that’s the purchasing power”* (P11 – Dentist) in addition to the advantage that access to sport clubs provides for children.

*“There are football, basketball and volleyball clubs. […] Within the city here we have higher, middle and lower social classes. The first two spheres allow their children to play there during holidays and within the school period”* (P19 – Licensed Nurse)

Contrary to preceding views that greater wealth equates to greater consumption of junk food, the prohibitive cost of healthy food was also thought to result in similar consequences for less wealthy families.

*“People here prefer to buy fast food because healthy food is expensive, they can’t buy fruits and vegetables, so they prefer to buy cheaper food although it isn’t healthy”* (P7 – Obstetrician)

### HCPs’ perceptions of their role in childhood obesity prevention and management

#### Educating parents about childhood obesity

In accordance with participants’ perceptions that parents have the most influence in childhood obesity and the perceived pervasiveness of the “*incorrect belief [that] a fat child is a healthy child”* (P9 – Surgeon) among parents, HCPs’ self-perception of their role predominantly focussed on educating parents as supporting the family unit was seen to be key to affecting a child’s behaviours.

*“[Encouraging healthy lifestyles in children] basically consists of educating the parents, doesn't it? Making parents aware of good nutrition for their children”* (P14 – Licensed Nurse)

*“We are here to support them, whether it is the parent or the family […] because that is our job, to support”* (P15 – Technical Nurse)

Addressing parental misconceptions was often central to the education provided by HCPs.

*“We advise the family, the parents, that it’s important to understand that their child is obese. It doesn’t mean that they are healthy, because there is a culture of this misconception that a fat child is a healthy child, and that is not true”* (P9 – Surgeon)

Promoting healthier lifestyles directly to parents was the mainstay of attempts to address obesity concerns as responsibility to initiate change was seen to be the parents’ domain. Educating the parents to teach their children healthy practices was the preferred long-term approach as the home was viewed as the source of learned behaviours.

*“Few children consume [healthy foods]. Parents don't teach them at home. Children learn from their environment, from home. We'll generally give them some ideas, some advice, but [unhealthy behaviours are] definitely from the home”* (P21 – Obstetrician)

Many HCPs acknowledged that their efforts to promote healthy lifestyles had little success, but most persisted for the few patients who benefitted from their advice.

*“We are here to guide the patient whether they understand or not, we are there every day to fight for the health of the whole family”* (P15 – Technical Nurse)

Conversely, one participant maintained that parents lacked education, but instead preferred to address this gap by targeting education towards the children themselves.

*“Unfortunately, parents aren’t interested, they aren’t educated. Or they already have the knowledge but for an adult to erase that is very difficult, it is already deeply embedded. That's why you always have to educate the little ones so, as they grow up, they will keep that knowledge”* (P13 – Licensed Nurse)

#### Regular monitoring of child growth

Generally, participants perceived monitoring growth in children to be a core aspect of a HCP’s role. At the two healthcare centres, measurements are routinely taken for all children who attend. Nurses appeared to hold this responsibility to a greater extent than other participants.

*“We, as workers, must be monitoring children whether they are obese or not […] it is our priority to keep the child healthy”* (P15 – Technical Nurse)

Routine monitoring of both weight and height enabled HCPs to recognise obese or overweight children and therefore created an opportunity to initiate interventions through a referral pathway to other HCPs.

*“Healthcare personnel are already dedicated to observing the weight and height of children […] Depending on their body mass they’re referred to the specialists, if they are obese, they’re automatically referred to the nutritionist so that the child can lose weight appropriately”* (P15 – Technical Nurse)

### Barriers and facilitators in childhood obesity prevention and management

#### Barriers and facilitators in healthcare

In the healthcare setting, a common barrier experienced by most participants was the lack of parental cooperation. This impacted HCPs’ ability to provide education and monitor child growth. Some felt that it was difficult to convince parents of the importance of childhood obesity because “*they always stick to their own beliefs about how to raise their children*” (P10 – Licensed Nurse) and thus were unlikely to comply with advice or return for further support.

*“There are some parents who do follow [advice], for example, five out of ten. The rest I think throw in the towel, they give up […] so they don't come back to their appointments”* (P20 – Licensed Nutritionist)

Many participants encountered difficulties explaining childhood obesity to parents due to a lack of engagement with services, such as missing follow-up appointments or not adhering to advice. However, none mentioned experiencing challenges related to raising the topic of obesity due to fear of stigma or offending parents.

*“I do everything possible so that the patient understands me. I practically have to scare them, because here we have to scare the parents and maybe then they’ll become aware that [obesity] can happen”* (P20 – Licensed Nutritionist)

Participants perceived that families only proactively consulted HCPs regarding weight concerns when they were already invested in cooperating with guidance. This included those who had encountered issues after ignoring previous healthcare advice.

*“There are mothers who only come once to the consultation and leave immediately then return after a few weeks when their child's health has already become complicated”* (P10 – Licensed Nurse)

Participants often praised the use of home visits to circumvent the lack of attendance at the healthcare centres. This enabled HCPs to contact families with the lowest attendance and therefore reach patients they believed to be of most concern.

*“We do home visits because the barrier was coming to the [child health] appointments”* (P6 – Family Doctor)

All comments on home visits were positive and many reported that this initiative was effective in terms of fulfilling their self-perceived duties in childhood obesity management and prevention which was to educate and monitor.

*“Our main role is to spread information to prevent illnesses, that is why we do home visits”* (P8 – Technical Nurse)

*“We have to do our job which is to follow-up on what [patients] are achieving, if they are succeeding or lack support, that is why home visits are made”* (P5 – Licensed Nurse)

#### Barriers and facilitators in schools

Overall, schools were viewed as an important source of health education for children and their parents. Some participants were part of long-standing programmes at schools to provide demonstrations regarding nutrition and portion sizes which they felt worked well.

*“Last year, we worked directly with the school and were able to train teachers and parents. The best intervention has been at the school”* (P21 – Obstetrician)

School-based interventions were presented as useful strategies when combined with other interventions, rather than as a stand-alone measure, as parental influences in the home remained most pertinent.

*“Don’t forget that schools are the second home of our children. They spend half of their life in school. So, if we have an enhancing effect between families and schools […] we could advance a lot”* (P9 – Surgeon)

Initiatives in schools appeared to be led mainly by HCPs as the participants perceived that “*in schools, even the teachers don’t care*” (P3 – Technical Nurse).

*“I think that if we, as healthcare professionals, don't go to the educational institutions they forget [about obesity] so we have to constantly advocate to strengthen the link with them”* (P11 – Dentist)

Therefore, schools were perceived to facilitate the HCPs’ aims by allowing them an additional platform to reach their patient population. However, the reluctant attitudes of teachers and parents still introduced barriers in this setting.

*“[Schools] always call parents in to talk about how they can have a healthy lifestyle with their children […] but the parents don't go, only a minority attend, […] so even though education is provided, I think more education should be given”* (P13 – Licensed Nurse)

Additionally, schools facilitated the main governmental policy that participants were aware of which centred around the introduction of healthier options at ‘kiosks’, stalls in schools where children buy lunch.

*“The ministry of health implemented a policy regarding healthy kiosks where school kiosks were required to sell healthy food such as fruit and salads, not the sweets that have typically been sold there”* (P9 – Surgeon)

Not all participants were aware of the healthy kiosks and many felt that the government was doing very little beyond this to prevent childhood obesity, which accorded with the HCPs’ own low level of concern for the issue.

*“The government is currently focusing on chronic childhood undernutrition […] the only thing being worked on [for childhood obesity] are the healthy lunches but not anything else”* (P14 – Licensed Nurse)

## Discussion

### Principal findings

Participants perceived that in Iquitos, childhood obesity was not a major concern as undernutrition was a greater priority. HCPs in this study framed obesity as a pathogenic condition with long-term adverse health effects but acknowledged that this view was not widespread. Instead, participants felt that the lay population believed overweight children were healthier. This recurrent theme informed participants’ views on their role and their attitudes towards parents. Although contextual factors related to Iquitos and socioeconomic status imparted some influence on the development of obesity in children, parental factors were commonly perceived to be the most influential as.

HCPs felt that parents lacked awareness regarding factors which contribute to childhood obesity such as health behaviours and beliefs. Education aimed at parents and, to a lesser extent, children in schools, were frequently presented as HCPs’ main strategy to prevent and manage childhood obesity. Additionally, monitoring child growth and accessing families in their homes were recognised by many participants as useful supplementary measures. Ultimately, most HCPs experienced difficulties impacting the issue of childhood obesity as they perceived the lack of parental cooperation to be an obstinate barrier which limited their capacity to affect meaningful change.

### Comparison with literature

The greater concern for undernutrition compared to childhood obesity reflects Loreto’s stage in the nutrition transition [10] as undernutrition remains highly prevalent in Peru’s rainforest regions [29]. The perceived influence of technology, and the importance of purchasing power in determining healthy lifestyles, accords with the notion that technological and economic development is important in the transition to an obesogenic environment [10, 30, 31]. The view that childhood obesity is not a priority in Iquitos, alongside the perception that contextual factors such as access to technology, healthy foods and outdoor space may be protective, has the potential to perpetuate even after these opinions cease to reflect reality. This may delay appropriate adaptation of strategies to negate increases in childhood obesity prevalence as observed elsewhere [20]. Therefore, local beliefs and factors should be continually reviewed to prevent HCPs from becoming complacent with their current low level of concern.

Participants were divided regarding the association between a family’s socioeconomic status and the likelihood of having obese children. A 2015 study of seven- and eight-year-olds in Peru found that obesity and overweight were associated with higher socioeconomic status in contradiction with findings from high-income countries [32], including those in Latin America [33]. However, obesity prevalence can shift from wealthier families to poorer families as economies develop [34] which may explain the HCPs’ mixed responses if Loreto is in the midst of this evolution. Therefore, the influence of socioeconomic status on childhood obesity trends requires further study to clarify these uncertainties.

Participants considered parents to be the most significant influence in the development of childhood obesity. HCPs perceived that most parents lacked sufficient education about obesity and struggled to engage with them. This is consistent with existing literature which established that HCPs experience difficulties involving parents in childhood obesity interventions [35]. Participants in this study did not experience challenges in communication resulting from beliefs that obesity was a sensitive topic which is contrary to previous findings [36–38]. Rather, HCPs noted that many parents lacked interest or motivation and successful consultations typically occurred with families already prepared to cooperate. These findings are corroborated by a qualitative study of paediatricians in the United States [39]. It has been found that HCPs often have minimal success managing childhood obesity despite adherence to guidelines, so studies recommend additional interventions in the wider community, such as in schools [39, 40]. Indeed, in 2013 Peru’s Ministry of Health implemented healthy school kiosks, as noted by a few participants, although the efficacy of these remain unclear [41–43]. Some participants did suggest greater collaboration with educational institutions which they felt were effective places to disseminate information. The perceived efficacy of school-based interventions is supported by a recent systematic review [44].

Participants’ self-perceived responsibility to monitor child growth is widely recognised as a key aspect of HCPs’ role in childhood obesity prevention [45, 46]. Measuring height to assess for undernutrition in children may receive greater attention in healthcare during the ‘dual burden’ stage of the nutrition transition but does not relay a child’s complete nutritional status [47]. However, participants in this study acknowledged the importance of monitoring weight, in addition to height, as part of child health assessments. This is a positive indication as it may facilitate recognition of the region’s evolving needs [47] and support HCPs to adjust their level of concern appropriately. Good surveillance practices can reveal initial changes in trends and thus aid prospective adaptation of nutrition policies which is critical to preventing the shift towards prevalent obesity in the early stages of the nutrition transition [20].

Many HCPs also advocated for the use of home visits to reach patients and spread information. This endorsement was related to the high rates of non-attendance that participants reported. Therefore, greater investment to increase capacity for home visits could address issues with uncooperative families, especially those who fail to attend appointments. This may also provide opportunities for home-based interventions targeted towards childhood obesity [48].

In this study, positive parental views of excess weight were an important barrier that HCPs faced and resonates with prior conclusions in this area of research [17, 49]. Existing literature recognises the prevalence of the parental perception that overweight children are healthy, particularly in Latin American mothers [50, 51]. This belief diminishes the likelihood of successful management of childhood obesity and necessitates further interventions to address the educational gap. In accordance with existing models for prevention and management strategies, participants suggested that greater parental involvement is required to improve the impact of childhood obesity interventions [52]. As shifting parental perceptions and enabling parents to recognise obesity or overweight in their child increases their intention to make health behaviour changes [53, 54], health visits to homes of unengaged families and frequent monitoring of all children should be continued to facilitate these. According to the HCPs’ perceptions in this study, increasing parental motivation to improve health behaviours and informing their health beliefs may be a potential target of childhood obesity interventions which is an implication for practice consistent with previous findings [55].

### Strengths and limitations

The qualitative nature of this study allowed individuals to raise unprompted topics which is particularly valuable in an area of research lacking literature from a similar setting. However, findings from this study should be cautiously interpreted as the 21 participants were self-selected from only two healthcare centres in Iquitos. It should be acknowledged that the lead researcher is an English-speaking university student and required an interpreter throughout recruitment and data collection. Cultural differences are known to influence cross-cultural research as the ‘outsider’ status of individuals conducting research in populations of which they are not a member may result in misinterpretations [56, 57]. However, this may also be a strength of the study as ‘outsider’ perspectives can provide valuable insights. Member validation was not sought and so conclusions drawn from the data may inaccurately represent participants’ views and, as HCPs with a duty to their patients, social-desirability bias may have affected responses. To minimise the impact of these factors, the voluntary nature of participation was clearly emphasised. Participants were also reassured that they could speak freely without judgement and would be anonymised.

## Conclusion

Considering the upward trend of childhood obesity in low and middle-income countries, the perceptions of HCPs in these settings are valuable to contextualise future initiatives. HCPs in this study perceived childhood obesity as a pathogenic issue but felt that this view was not shared by parents in Iquitos, where childhood obesity prevalence rates are relatively low. Without timely intervention, Iquitos is likely to complete the nutrition transition as experienced in Peru’s coastal regions. To prevent this, findings from this study can help to inform the development of targeted public health strategies which are sensitive to local contexts.

## Supplementary Information

**Additional file 1.** Topic Guide.

## Data Availability

Data are available from the corresponding author on reasonable request.

## Abbreviation

HCP: Healthcare professionals
