# Supplementary material for: Healthcare professionals’ perceptions of childhood obesity in Iquitos, Peru: a qualitative study
Source: BMC Health Serv Res. 2022 Feb 10;22:175. doi: 10.1186/s12913-022-07519-z (PMC8832769; doi:10.1186/s12913-022-07519-z)
Supplement: Supplementary file 1 — Additional file 1. Topic Guide. [file 12913_2022_7519_MOESM1_ESM.docx]

# Additional file 1: Topic Guide

**Introduction**

Hello, my name is [insert researcher’s name] and I am a medical student from the University of Birmingham in the United Kingdom. Thank you very much for agreeing to take part in this study. Please can you confirm that you have the read the participant information sheet and that you consent be a participant.

**Questions**

I am now going to ask you some questions about childhood obesity, you are free to respond however you wish and can skip a question or stop the interview altogether.

| **Objectives** | **Questions and optional prompts** |
| --- | --- |
| **Perceptions and attitudes towards childhood obesity** | What do you understand about childhood obesity?   - Is it a problem? - Is it increasing?   What are the implications of being overweight or obese as a child?   - How does it impact on different aspects of a child’s development or upbringing (for example in terms of health, social, emotional, financial etc. aspects)? - What are the short-term consequences? - What are the long-term consequences?   What constitutes a healthy lifestyle?   - Particularly in children? - Is it difficult to follow a healthy lifestyle? - What are the consequences of an unhealthy lifestyle? |
| **Factors which healthcare professionals perceive to be important in the development of childhood obesity** | What factors do you think impact childhood obesity?   - What do you think about children’s understanding of obesity? - How do children’s friends and family influence healthy behaviours and the development of obesity? - What role do schools; healthcare centres and childcare centres (or other institutions) have in influencing childhood obesity trends? - What role do different communities or groups (religious groups, ethnic groups, neighbourhoods) have in influencing childhood obesity trends? - What role do government policies have in influencing childhood obesity trends?   What are your views on how these factors influence childhood obesity?   - Positive influence? - Negative influence? |
| **Perceptions of healthcare professionals’ role in childhood obesity prevention and management** | What role do you think healthcare professionals have in childhood obesity?   - How important is your role/the role of healthcare professionals in childhood obesity? - What roles do you think are (the most) important? - How does the role of healthcare professionals compare to other factors which might influence childhood obesity?   How much impact do healthcare professionals have on children and obesity?   - In your experience, in what ways can you impact childhood obesity trends? - What is your view of how much impact your role has in childhood obesity? - How much impact do healthcare professionals have in influencing children’s attitudes and behaviours? - How much impact do healthcare professionals have in influencing the activities of institutions? - How much impact do healthcare professionals have in influencing the beliefs and attitudes about childhood obesity amongst different communities? - How much impact do healthcare professionals have in influencing policy at a local and national level? |
| **Perceptions of barriers and facilitators in childhood prevention and management** | What is your understanding of prevention and management in childhood obesity?   - What does prevention involve? - What does management involve?   What barriers are there to preventing and managing childhood obesity?   - To understanding childhood obesity - To discussing the topic with children and their parents - To initiating prevention or management strategies   What facilitators are there to preventing and managing childhood obesity?   - To understanding childhood obesity - To discussing the topic with children and their parents - To initiating prevention or management strategies |

**Closing remarks**

Those are all the questions I have for this interview. Do you have anything else to add about anything we have covered or a topic I have not mentioned yet?

**End the interview**

Thank you for taking part. The interview has now finished. If you have any questions, please feel free to get in contact with me or the local supervisor through the details on the participant information sheet. If you would like to withdraw from the study, you can do so in the next three days. After this time, your responses will be anonymised.

[Turn off the audio recorder, collect paperwork and thank participant]

**Reflection and write-up of field notes**

How did the interview go?

What were the main issues discussed?

Did new topics arise?

Does the topic guide need to be adapted?

How does this interview compare to other data?
